# Topological Dimensions from Disorder and Quantum Mechanics?

**DOI:** 10.3390/e25111557

**Published:** 2023-11-17

**Authors:** Ivan Horváth, Peter Markoš

**Affiliations:** 1 Nuclear Physics Institute CAS, 25068 Řež near Prague, Czech Republic; 2 Department of Physics and Astronomy, University of Kentucky, Lexington, KY 40506, USA; 3 Department of Experimental Physics, Faculty of Mathematics, Physics and Informatics, Comenius University in Bratislava, Mlynská Dolina 2, 842 28 Bratislava, Slovakia; peter.markos@fmph.uniba.sk

**Keywords:** Anderson transition, localization, effective counting dimension, effective number theory, effective support, dimension content, emergent space

## Abstract

We have recently shown that the critical Anderson electron in D=3 dimensions effectively occupies a spatial region of the infrared (IR) scaling dimension $d_{\mathrm{IR}}\;\approx \;8/3$. Here, we inquire about the dimensional substructure involved. We partition space into regions of equal quantum occurrence probabilities, such that the points comprising a region are of similar relevance, and calculate the IR scaling dimension *d* of each. This allows us to infer the probability density p(d) for dimension *d* to be accessed by the electron. We find that p(d) has a strong peak at *d* very close to two. In fact, our data suggest that p(d) is non-zero on the interval $\left[d_{\min},d_{\max}\right]\;\approx \;\left[4/3,8/3\right]$ and may develop a discrete part (δ-function) at d=2 in the infinite-volume limit. The latter invokes the possibility that a combination of quantum mechanics and pure disorder can lead to the emergence of integer (topological) dimensions. Although $d_{\mathrm{IR}}$ is based on effective counting, of which p(d) has no a priori knowledge, $d_{\mathrm{IR}}\;\geq \;d_{\max}$ is an exact feature of the ensuing formalism. A possible connection of our results to the recent findings of $d_{\mathrm{IR}}\;\approx \;2$ in Dirac near-zero modes of thermal quantum chromodynamics is emphasized.

## 1. Introduction

Understanding the spatial geometry of Anderson transitions [1] is an intriguing problem. Indeed, although studied quite extensively, the complicated structure of critical electronic states (see e.g., [2]) leaves room for new insights. Novel characterization may reveal unknown details of disorder-driven metal-insulator transitions and, for example, lead to a deeper understanding of their renormalization group description [3].

Another reason to study the geometry of Anderson transitions arises by seeing them as quantum *dimension transitions*, a viewpoint taken in Ref. [4]. Using effective number theory (ENT) [5,6], which entails a unique measure-based dimension $d_{\mathrm{IR}}$ [7,8] for spaces with probabilities, it was shown there that the transition is a two-step dimension reduction

(1) $$d_{\mathrm{IR}}\;=\;3\;\;⟶\;\;\;\approx 8/3\;\;⟶\;\;0$$

Here the flow is from the extended to critical to localized states, and exponential localization was assumed. A remarkable property of the above is that these reductions are complete [9]. Indeed, the probability does not leak away from subdimensional effective supports, and the electron is fully confined to them in infinite volume. It is thus meaningful to say that the space available to quantum particles collapses into a lower-dimensional one under the influence of strong enough disorder. As such, it represents a mechanism for generating lower-dimensional spaces by simple combination of quantum mechanics and disorder.

While dimension is the most basic characteristic of space available to a critical electron, this space may contain subsets with dimensions $d\;<\;d_{\mathrm{IR}}$. Such a substructure may be physically significant if electron mostly resides there. The aim of this work is to characterize the critical spatial geometry in such a manner: we will compute the probability distribution p(d) that the electron is present in a space of dimension *d*. We refer to p(d) as the *dimension content* of the Anderson criticality or that of the probability distribution in general.

Critical states at Anderson transitions were recognized to have fractal-like features long ago, first interpreting them in analogy to scale-invariant fractals [10,11] and, later, to more complex multifractals [12,13,14,15]. The formalism used in the latter mimics one that describes the ultraviolet (UV) measure singularities occurring in turbulence and strange attractors (see, e.g., [16,17]). More recent works in the Anderson context are [18,19,20,21,22]. However, the focus of multifractal analysis does not make it convenient for computing p(d). We thus proceed by proposing a method that organizes the calculation in terms of probabilities from the outset and zooms in on dimensions by the degree of their actual presence. Moreover, the *d* involved is simply the IR Minkowski dimension of a subset and thus manifestly a measure-based dimension of space. In the ensuing multidimensionality formalism, a given wave function is

(2) $$\begin{array}{cc} \mathrm{subdimensional}\;\mathrm{if} & \;\;d_{\mathrm{IR}}<D\;\\ \mathrm{multidimensional}\;\mathrm{if} & \;\;p\left(d\right)\neq \delta \left(d-d_{\max}\right)\;\\ \mathrm{of}\;\mathrm{proper}\;\mathrm{dimension}\;\mathrm{if} & \;\;d_{\mathrm{IR}}=d_{\max} \end{array}$$

where $d_{\max}\;=\;\sup\;\left\{d\;|\;p\left(d\right)>0\right\}$, D=3 is the IR dimension of the underlying space, and $d_{\mathrm{IR}}\geq d_{\max}$ holds in general.

**Figure 1:**
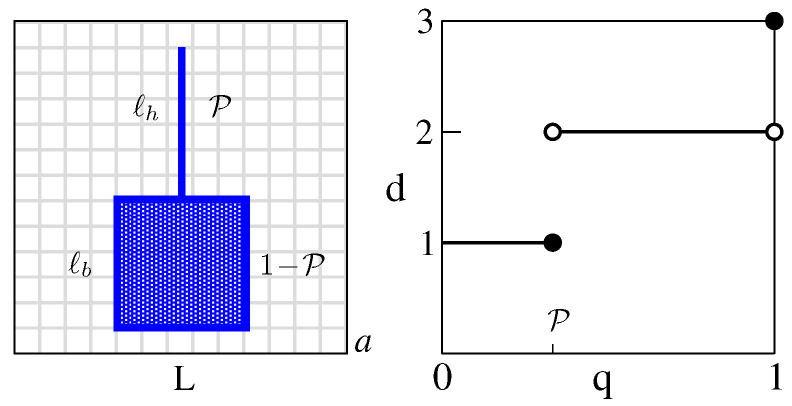
The “shovel” (**left**) and d(q) (**right**) associated with its UV dimension content in $R^{3}$. See the discussion in the text.

Before proceeding to define p(d), we illustrate the idea on a “shovel” in $R^{D\;=\;3}$ space (Figure 1). The shovel consists of 2D square blade and 1D handle with the uniformly distributed masses $M_{b}\;>\;0$ and $M_{h}\;>\;0$, respectively. If the relevance of space points is set by the mass they carry, the probabilities of encountering the handle, the blade, and the rest of space are $P=M_{h}/\left(M_{b}\;+\;M_{h}\right)$, 1−P and 0, respectively. Note that the UV cutoff *a* and IR cutoff *L* are also indicated.

Above, we implicitly assumed that *d* is the usual UV dimension (a→0 at a fixed *L*), in which case we have by inspection p(d)=Pδ(d−1)+(1−P)δ(d−2). However, how would this p(d) be concluded by a computer that cannot “see” and only processes regularized probability vectors $P\left(a\right)\;=\;\left(p_{1},p_{2},\ldots ,p_{N(a)}\right)$? Here, $N\left(a\right)\;=\;{\left(L/a\right)}^{3}$, $p_{i}$ is the probability within an elementary cube at the point $x_{i}$ of a latticized space, and a∈{L/k∣k=2,3,…}.

Anticipating that any number *J* of discrete dimensions $0\;\leq \;d_{1}\;<\;d_{2}\;<\;\ldots <d_{J}\;\leq \;3$ with probabilities $P_{j}\;>\;0$ could be present, the computer first orders $p_{i}$ in each P(a) so that $p_{1}\;\geq p_{2}\;\geq \ldots \geq p_{N(a)}$. The rationale is that, with decreasing *a*, this increasingly better separates out populations related to a different $d_{j}$. Indeed, the typical size of *p* associated with $d_{j}$ is $\propto \;a^{d_{j}}$ and so P(a) gradually organizes into *J* sequential blocks starting with $d_{1}$. The above ordering in *P* will always be assumed from now on.

To detect possible blocks/dimensions, the computer uses the variable q∈[0,1] for cumulative probability, and associates with each P(a) the function ν(q,a), namely the number of first elements in P(a) (space points) whose probabilities add up to *q*. Keeping track of the fractional boundary contributions at each *q* makes it a continuous, convex, increasing, piecewise linear function such that ν(0,a)=0 and ν(1,a)=N(a). The number of points in the interval (q−ϵ,q] is ν(q,a)−ν(q−ϵ,a) and scales as $a^{-d(q,\epsilon )}$ for a→0. When processing P(a) for the shovel, the computer finds perfect scaling $(\ell_{h}/a)\times \epsilon /P$ for ϵ≤q≤P, and ${\left(\ell_{b}/a\right)}^{2}\times \epsilon /\left(1-P\right)$ for P+ϵ<q<1. It will thus conclude d(q) shown in Figure 1 upon ϵ→0. The value at q=1 represents the spatial complement of the shovel (zero probability). Collecting the probability of *d*, namely $p\left(d\right)=\int_{0}^{1}dq\;\delta \left(d-d\left(q\right)\right)$, produces the inspected result.

Two points are relevant here. (1) The above approach does not change if a continuous set of dimensions is present. In this case the obtained d(q) is not piecewise-constant but rather a piecewise-continuous, non-decreasing function, possibly with constant parts identifying discrete dimensions. (2) The IR case is fully analogous, but it is useful to recall the meaning of the IR dimension (L→∞, *a* fixed) which is somewhat non-standard. Thus, if both $\ell_{h}$ and $\ell_{b}$ are fixed as L→∞ (the usual case), then p(d)=δ(d) since the populations at each *q* remain constant. However, if e.g., $\ell_{b}$ is fixed while the handle responds by $\ell_{h}\propto L$ (the shovel reaches anywhere in space), then p(d)=(1−P)δ(d)+Pδ(d−1).

## 2. The Formalism

We now define p(d) in the IR setting of the Anderson transitions. Such analysis pertains to the wave functions $\psi \;=\;\psi (r_{i})$ on a cubic lattice of $N\left(L\right)\;=\;{\left(L/a\right)}^{D}$ sites $r_{i}$, with *L* the IR regulator and *a* set to unity. With ψ, we associate the probability vector $P\;=\;(p_{1},p_{2},\ldots ,p_{N=N(L)})$, where $p_{i}=\psi^{+}\psi \left(r_{i}\right)$, the effective number of sites [5,6]

(3) $$N_{★}\left[\psi \right]\;=\;\sum_{i=1}^{N}n_{★}\left(Np_{i}\right)\;,\;n_{★}\left(c\right)\;=\;\min\;\left\{c,1\right\}\;$$

and the cumulative count ν[q,ψ] defined as follows. Consider the cumulative probabilities $\left(q_{0},q_{1},\ldots ,q_{N}\right)$ with $q_{0}\;=\;0$ and $q_{j}\;=\;\sum_{i=1}^{j}p\left(i\right)$ for j>0. Let j(q), q∈(0,1) be the largest *j* such that $q_{j}\;<\;q$. Then ν[0,ψ]=0, ν[1,ψ]=N and

(4) $$\nu \left[q,\psi \right]=j\left(q\right)+\frac{q-q_{j}}{q_{j+1}-q_{j}}\;\;,\;\;0<q<1\;\;$$

Recalling the order in *P*, ν[q,ψ] is increasing and convex.

Consider the Anderson model in the orthogonal class [1]. With $c_{r_{i}}$, the electron operators, the Hamiltonian is

(5) $$H\;=\;\sum_{i}\epsilon_{r_{i}}\;c_{r_{i}}^{†}\;c_{r_{i}}\;+\;\sum_{i,j}c_{r_{i}}^{†}\;c_{r_{i}-e_{j}}+h.c.$$

where $e_{j}$ (j=1,...,D) are unit lattice vectors and random potentials $\epsilon_{r_{i}}\;\in \;\left[-W/2,+W/2\right]$ are uniformly distributed. The physics of the model involves averaging over disorder $\left\{\epsilon_{r_{i}}\;\right\}$. For $N_{★}$ and ν of one-particle eigenstates ψ at an energy *E*, we have

(6) $$N_{★}\left[\psi \right]\to N_{★}\left(E,W,L\right)\;\;\;,\;\;\;\nu \left[q,\psi \right]\to \nu \left(q,E,W,L\right)\;\;$$

Keeping the dependence on *E* and *W* implicit, the L→∞ behavior defines the dimensional characteristics $d_{\mathrm{IR}}$ and d(q) via

(7) $$N_{★}\left(L\right)\;\propto \;L^{d_{\mathrm{IR}}}\;\;\;,\;\;\;\nu \left(q,L\right)-\nu \left(q-\epsilon ,L\right)\;\propto \;L^{d(q,\epsilon )}\;\;\;$$

with $d\left(q\right)\;=\;\lim_{\epsilon \to 0}d\left(q,\epsilon \right)$. Due to the convexity of cumulative counts, d(q,ϵ) and d(q) are non-decreasing. The probability density of finding the IR dimension *d* in a state is then

(8) $$p\left(d,\epsilon \right)=\int_{0}^{1}dq\;\delta \left(d-d(q,\epsilon )\right)\;\;\;,\;\;\;p\left(d\right)=\lim_{\epsilon \to 0}p\left(d,\epsilon \right)\;\;$$

If d(q) is differentiable at *q*, then $p\left(d\;=\;d\left(q\right)\right)\;=\;1/d^{\prime }\left(q\right)$. The range of d(q), equal to the support of p(d), specifies the IR dimensions occurring with non-zero probability in states of interest. It is a subset of $\left[d_{\min},d_{\max}\right]$ where

(9) $$d_{\min}\;=\;\inf\left\{d|p\left(d\right)\;>\;0\right\}\;\;,\;\;d_{\max}\;=\;\sup\left\{d|p\left(d\right)\;>\;0\right\}\;$$

Important feature of the ensuing formalism is that

(10) $$d_{\max}\leq d_{\mathrm{IR}}\leq D\;$$

Here, the inequalities involving *D* are obvious and the last one can be most easily seen in discrete cases. Indeed, let $p\left(d\right)\;=\;\sum_{j=1}^{J}P_{j}\delta \left(d\;-\;d_{j}\right)$ with $0\;\leq \;d_{1}\;<\;\ldots \;<\;d_{J}\leq D$, $P_{j}\;>\;0$, and assume that $d_{\mathrm{IR}}\;<\;d_{J}\;=\;d_{\max}$. Consider *q* such that $1\;-\;P_{J}\;<\;q\;<\;1$. Then, $\nu \left(q,L\right)\;-\;\nu \left(q-\epsilon ,L\right)\;=\;\epsilon \;v\left(q,\epsilon ,L\right)L^{d(q,\epsilon )}$ for sufficiently small ϵ, where $\lim_{\epsilon \to 0}d\left(q,\epsilon \right)\;=\;d_{J}$ and $\lim_{\epsilon \to 0}\lim_{L\to \infty }v\left(q,\epsilon ,L\right)\;=\;v\left(q\right)\;>\;0$. The size of the individual p=ϵ/(ν(q,L)−ν(q−ϵ,L)) in this population is then $L^{-d(q,\epsilon )}/v\left(q,L,\epsilon \right)$. Hence, if $d_{J}\;<\;D$, then min{1,Np} in the definition of $N_{★}$ yields one for a sufficient *L* and ϵ, while if $d_{J}\;=\;D$, it yields 1/v(q). In both cases, the contribution of this population to $N_{★}$ is $\propto \;L^{d_{J}}$. Hence, $d_{\mathrm{IR}}\geq d_{J}$, which contradicts the assumption and leads to (10).

## 3. Anderson Criticality

We now perform the dimensional analysis for critical states of D=3 Anderson Hamiltonian (5) with periodic boundary conditions at the critical point $\left(E_{c},W_{c}\right)\;=\;\left(0,16.543\left(2\right)\right)$ [23]. The calculations in Ref. [4] yielded $d_{\mathrm{IR}}\;=\;2.665\left(2\right)\;\approx \;8/3$. For d(q) we follow [4], keeping track of dimension defined at a finite *L* and extrapolating it directly. In particular,

(11) $$d\left(q,\epsilon ,L\right)=\frac{1}{\log s}\;\log\frac{\nu (q,L)-\nu (q-\epsilon ,L)}{\nu (q,L/s)-\nu (q-\epsilon ,L/s)}\;\;\;$$

with fixed s>1, and $d\left(q,\epsilon \right)\;=\;\lim_{L\to \infty }d\left(q,\epsilon ,L\right)$. In the analysis, we set s=2. For 34 sizes in the range 16≤L≤144, two near-zero eigenmodes were computed at 40k–100k disorder samples using the JADAMILU package [24]. We set $\epsilon \;=\;10^{-3}$, thus splitting the interval q∈[0,1] into 1000 bins and evaluating $d(q_{b},\epsilon ,L)$ at $q_{b}=b\times 10^{-3}$, b=1,…,1000. We verified that this is fine enough to directly represent the ϵ→0 limits for our purposes.

**Figure 2:**
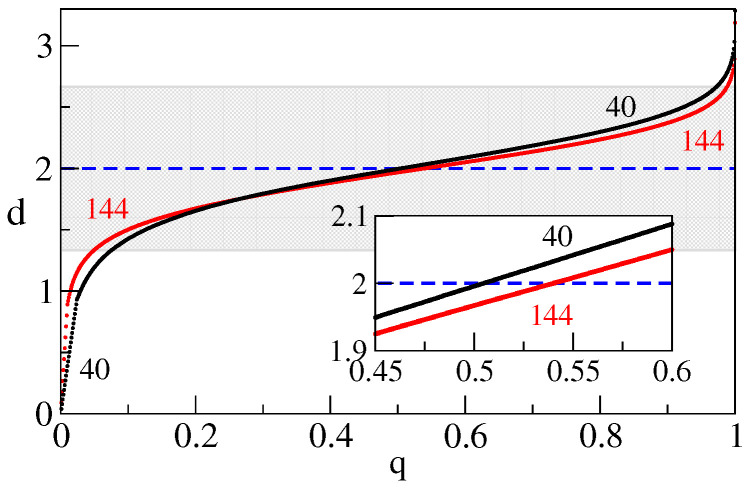
Function d(q,ϵ,L) at $\epsilon \;=\;10^{-3}$ for L=40 and L=144 (largest) systems. Shaded region marks the range d∈[4/3,8/3].

Given that, we show d(q,L) at L=40 and L=144 in Figure 2. An important feature of the obtained behavior is the flatness in the middle part of *q*, indicating large probabilities for dimensions in the corresponding range. An increase of *L* results in a flatter d(q,L) and yet a sharper range of prominent dimensions. The visible linear parts at small *q* mark regions where finite-size effects lead to non-convex ν(q). Their extent shrinks toward zero with growing *L*. Linearity was imposed to keep the behavior regular.

**Figure 3:**
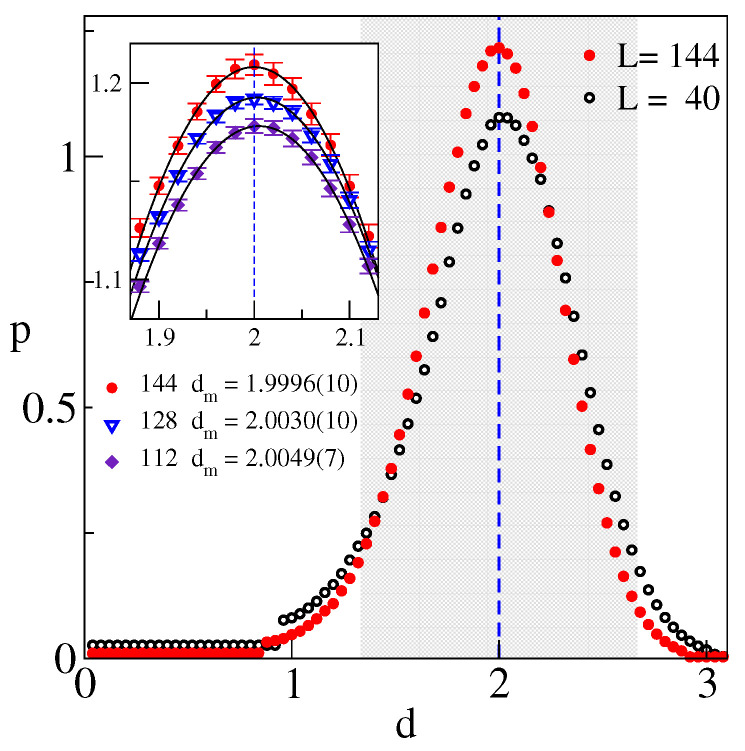
Function p(d,ϵ,L) at $\epsilon \;=\;10^{-3}$ for L=40 and L=144 (largest) systems. Shaded region marks the range d∈[4/3,8/3].

The corresponding p(d,L) obtained via (8) are shown in Figure 3. We observe sharp peaks of decreasing width, centered at $d_{m}\;\approx \;2$. The error bars, too small to be visible, were obtained via the Jackknife procedure with respect to disorder samples. The stability of $d_{m}$ and its proximity to 2 is quite remarkable, as shown in the inset for the largest sizes studied. The quoted values were obtained from quadratic fits in the displayed vicinity of the maximum. The constant parts at a small *d* correspond to the linear segments in Figure 2.

**Figure 4:**
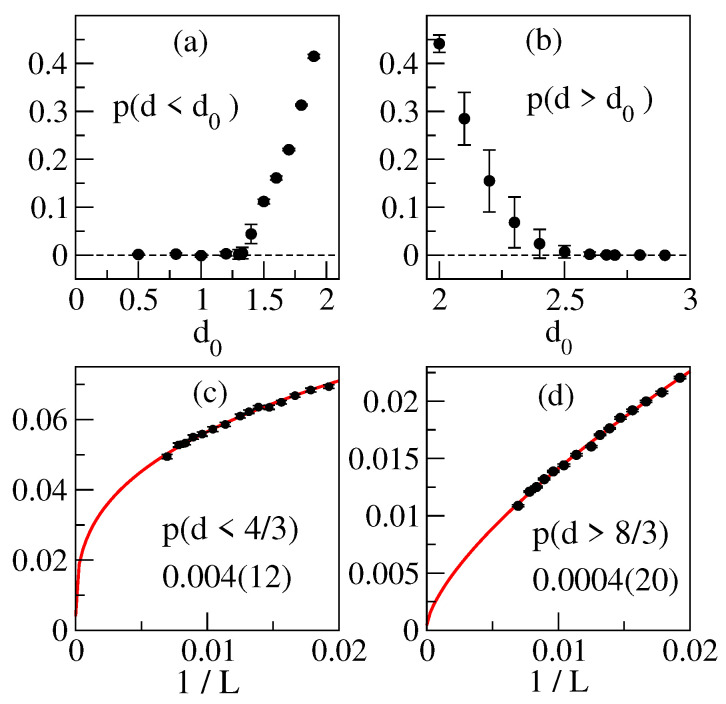
Probabilities $p(d\;<\;d_{0})$ and $p(d\;>\;d_{0})$ (panels (**a**,**b**)) in L→∞ limit. Panels (**c**,**d**) show extrapolations for $d_{0}\;=\;4/3$ and $d_{0}\;=\;8/3$.

Among the key chracteristics of the dimension content p(d) is its support, i.e., dimensions that can contribute to physical processes with non-zero probability density. The above properties of p(d,L) imply that the support in fact spans certain $\left[d_{\min},d_{\max}\right]$, and its specification thus reduces to finding $d_{\min}$ and $d_{\max}$. To that effect, we evaluate the probabilities $p(d\;<\;d_{0},L)$ of dimensions smaller than $d_{0}$ and vary $d_{0}$ upward. For each $d_{0}$, $p(d\;<\;d_{0},L)$ is the L→∞ extrapolated by fitting to a constant with general power correction. The result, shown in Figure 4 panel (a), features a probability threshold turning on near $d_{0}\;=\;1.3$. We take $d_{0}\;=\;4/3$ as a reference value: in panel (c), we show its extrapolation leading to a clean statistical zero. The analogous procedure based on $p(d\;>\;d_{0})$ yields the results shown in panels (b) and (d) with $d_{0}\;=\;8/3$ referencing the other threshold.

**Figure 5:**
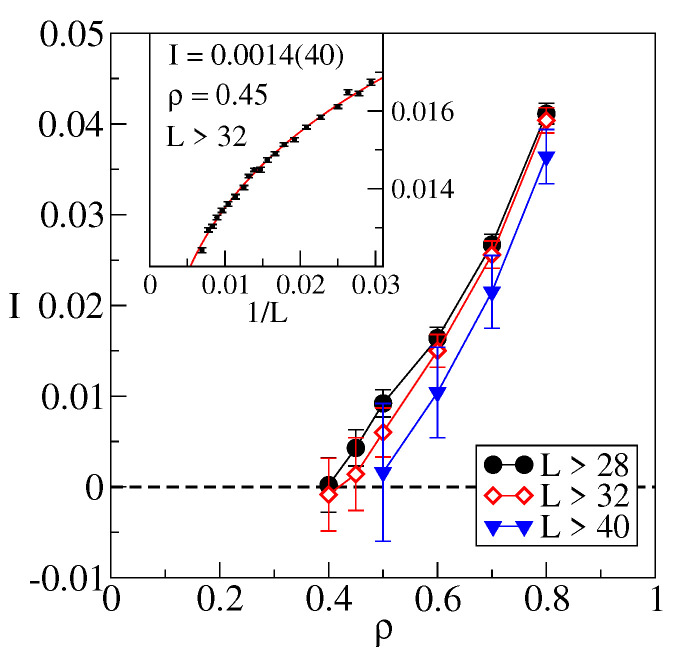
Function I(ρ,L→∞) obtained by fitting in *L*-ranges containing increasingly larger lattices. Inset shows example of a fit in the vicinity of $\rho_{0}$ such that $I(\rho_{0})\;\approx \;0$.

Given the strong dominance of $d_{m}$, the second key question is whether $d_{m}$ could be a discrete dimension present in Anderson critical states. This would mean that, in the L→∞ limit, d(q,L) (see Figure 2) develops a strictly constant part in certain range of *q*. We will test this possibility for the observed $d_{m}\;=\;2$ via the following procedure. Given a d(q,L), we find $q_{2}\left(L\right)$ such that $d(q_{2},L)=2$ and calculate

(12) $$I\left(\rho ,L\right)=\int_{q_{2}-\rho /2}^{q_{2}+\rho /2}dq\;(\;2-d(q,L)\;{)}^{2}$$

which is only zero if d(q,L)=2 on the interval. For a given ρ, we perform the L→∞ extrapolation via fit to a constant I(ρ) with general power correction. Fitting data for systems with L>28 leads to the results shown in Figure 5 (circles). Notice a steep decay of I(ρ) with decreasing ρ, reaching I≈0 at ρ≈0.4 with errors becoming large below this point. While this could simply indicate a very steep analytic behavior of I(ρ), further analysis suggests otherwise. Indeed, restricting fits to larger systems, namely L>32 (diamonds) and L>40 (triangles), results in an increasingly steeper decay toward zero at yet larger ρ. The natural interpretation of these tendencies is that I(ρ)≡0 for $\rho \;<\;\rho_{0}\;\approx \;0.5$, pointing to the discrete nature of $d_{m}$.

**Figure 6:**
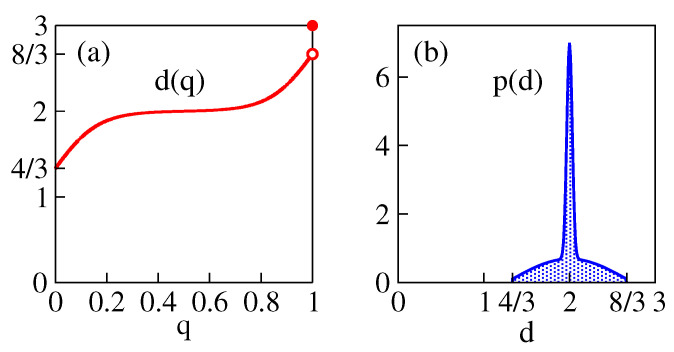
Schematic representation of the concluded function d(q) (panel (**a**)) and the dimensional content p(d) (panel (**b**)) at Anderson criticality. The narrow spike in (**b**) represents the δ-function.

The synthesis of our results suggests the following form of the spatial dimension content at the Anderson criticality

(13) $$p\left(d\right)=P\;\delta \left(d-d_{m}\right)\;+\;\left(1-P\right)\;\pi \left(d\right)$$

where π(d) is a continuous probability distribution with support on the interval $\left[d_{\min},d_{\max}\right]$. The parameters are

(14) $$d_{m}\;\approx \;2\;\;\;,\;\;\;d_{\min}\;\approx \;4/3\;\;\;,\;\;\;d_{\max}\;\approx \;8/3\;\;\;,\;\;\;P⪆1/2$$

where we estimate the accuracy of $d_{m}$ at the couple ‰ and that of $d_{\min}$, $d_{\max}$ at the couple %. The graphical representation of this result in terms of d(q) and p(d) is shown in Figure 6.

## 4. Discussion

We proposed that, in addition to their measure-based effective dimension ($d_{\mathrm{UV}}$ or $d_{\mathrm{IR}}$) [5,6,7,8], probability distributions on metric spaces can be characterized by the associated *dimension content* p(d). The method was applied to the structure of critical states in the D=3 Anderson transition (O class). Here, p(d) identifies the dimensions of regions in which the electron can in fact be found, i.e., those relevant to its physics. Critical wave functions are subdimensional, multidimensional, and our new results are summarized by Equations (13) and (Section 3). A few comments should be made.

(i) The picture of the Anderson transition as a spatial dimension transformation (1) receives key refinements by virtue of p(d). Indeed, although the critical electron is fully confined to the spatial effective support $S_{★}$ of Minkowski dimension $d_{\mathrm{IR}}\;\approx \;8/3$ [4,9], its key substructure has $d_{m}\;\approx \;2$, and the continuum of lower- and higher-dimensional features is also present. Geometrically, $S_{★}$ may thus also be viewed as a surface-like structure endowed with complex lower-dimensional “hair” and higher-dimensional “halo”.
(ii) Our results suggest that $d_{m}$ is a discrete dimension and that it may assume an exact topological value of $d_{m}\;=\;2$. [The mathematical meaning of “topological” in the context of IR dimension would, of course, need some clarification.] This invokes a possibility that quantum mechanics combined with pure disorder can lead to the emergence of integer dimensions. Apart from an understanding of Anderson transitions, variations on such dynamics could find relevance in modeling an emergent space in the early universe. A more detailed description of this geometry would be needed.
(iii) The connection between $d_{\mathrm{IR}}$ and p(d) results from the built-in additivity that makes both measure-based: in the case of $d_{\mathrm{IR}}$ it is the additivity of effective counting with respect to combining the systems [5,6], and in the case of d(q) the familiar additivity of ordinary counting. This aspect is key to the interpretation of these concepts as spatial dimensions. Indeed, it is because the Hausdorff measure and the Minkowski count properly quantify volume that dimensions based on them became useful and accepted characteristics of space.
(iv) It is natural to ask whether some features of the described spatial structure have analogues in the multifractal approach [16,17] adopted to the IR Anderson setting via the moment method [25]. Here the focus is on the so-called dimensional spectrum f(α). Inner workings of the method give special status to the information dimension [26] in a way somewhat similar to $d_{m}$. It would be interesting to study the possible association between the two in detail. (See also the debate regarding $d_{\mathrm{IR}}$ in Refs. [27,28,29].)
(v) Our data are consistent with critical wave functions being of proper dimension ($d_{\mathrm{IR}}\;=\;d_{\max}$). However, albeit state of the art, their statistical power is not sufficient to reach a sharper conclusion at this point.
(vi) Our findings acquire another angle in light of recent results [7,30] in quantum chromodynamics (QCD). The original proposal that the Anderson-like mobility edge $\lambda_{A}\;>\;0$ appears in the QCD Dirac spectrum upon thermal chiral transition [31,32], worked out by Refs. [33,34,35], became more structured. Indeed, the existence of a new mobility edge $\lambda_{\mathrm{IR}}\;\equiv \;0$ has been concluded, and its simultaneous appearance with $\lambda_{A}$ at temperature $T_{\mathrm{IR}}$ was conjectured [30]. Here $T_{\mathrm{IR}}$ marks the transition to a phase featuring the IR scale invariance of glue fields [36]. The approach to IR criticality ($\lambda \;\to \;\lambda_{\mathrm{IR}}^{+}$) was found to proceed via $d_{\mathrm{IR}}\;\approx \;2$ Dirac modes [7], with the topological origin of the dimension suspected. Clarifying a possible relation of this to $d_{m}\;\approx \;2$ found here may shed new light on the QCD–Anderson localization connection.
(vii) The proposed IR/UV guises of multidimensionality formalism easily extend to more general situations without the metric. Here the sequence $\left\{O_{k}\right\}$ involving collections $O_{k}\;=\;\left(o_{k,1},o_{k,2},\ldots ,o_{k,N_{k}}\right)$ with an increasing number $N_{k}$ of arbitrary objects comes with the associated sequence $\left\{P_{k}\right\}$ of the relevance (probability) vectors. The role of $d_{\mathrm{IR}}$ and $d_{\mathrm{UV}}$ is taken by the effective counting dimension 0≤Δ≤1 defined via scaling $N_{★}\left[P_{k}\right]\propto N_{k}^{\;\Delta }\;\mathrm{for}\;k\to \infty$ [8]. The dimension function d(q) is replaced by an analogous γ(q) and the dimension content p(d) by p(γ). The target (k→∞) effective collection defined by $\left\{O_{k}\right\}$, $\left\{P_{k}\right\}$ is then
  (15) $$\begin{array}{cc} \mathrm{subdimensional}\;\mathrm{if} & \;\;\Delta <1\;\\ \mathrm{multidimensional}\;\mathrm{if} & \;\;p\left(\gamma \right)\neq \delta \left(\gamma -\gamma_{\max}\right)\;\\ \mathrm{of}\;\mathrm{proper}\;\mathrm{dimension}\;\mathrm{if} & \;\;\Delta =\gamma_{\max} \end{array}$$
  where $\gamma_{\max}=\sup\;\left\{\gamma \;|\;p\left(\gamma \right)>0\right\}$ and $\gamma_{\max}\leq \Delta$.

## Data Availability

The numerical data presented here is available upon request.
